# Precision of a phage susceptibility spot assay assessed with 154 clinical *Staphylococcus aureus* isolates

**DOI:** 10.1128/spectrum.03284-25

**Published:** 2026-03-03

**Authors:** Sebastian C. Herren, Christoph L. Zink, Jay Mandrekar, Robin Patel

**Affiliations:** 1 Division of Clinical Microbiology, Department of Laboratory Medicine and Pathology, Mayo Clinic 6915, Rochester, Minnesota, USA; 2 Division of Clinical Trials and Biostatistics, Department of Quantitative Health Sciences, Mayo Clinic Rochester 4352 , Rochester, Minnesota, USA; 3 Division of Public Health, Infectious Diseases and Occupational Medicine, Department of Medicine, Mayo Clinic 6915, Rochester, Minnesota, USA; University of Maryland School of Medicine, Baltimore, Maryland, USA

**Keywords:** Methicillin-resistant *Staphylococcus aureus *(MRSA), phage K, phage susceptibility testing, phage therapy

## Abstract

Lytic phages are a promising consideration to combat recalcitrant bacterial infections. To advance the possibility of using phage therapy in the clinical routine, standardized phage susceptibility testing methods are needed. Here, staphylococcal phage K was tested against 154 periprosthetic joint infection-associated *Staphylococcus aureus* isolates using a double overlay spot assay in technical and biological triplicates. Individual plaques were assessed using a morphology scoring system ranging from 0 (no plaques) to 4 (large, clear plaques), with scores ≥2 considered indicative of infectivity. Overall, 107 isolates exhibited scores ≥2. The intra-day and inter-day coefficients of variation were 0.9 and 2.5, respectively. Plaque-forming unit per milliliter (PFU/mL) titers correlated with morphology scores (Spearman’s ρ = 0.8, *P* < 0.0001). The results suggest high precision of the described spot assay assessing *S. aureus* susceptibility to phage K.

**IMPORTANCE:**

Standardized laboratory assays for assessing phage susceptibility are a prerequisite for clinical implementation of phage therapy. No assay has been denoted a reference standard. Criteria that can be used to assess assay precision and suitability for routine testing are intermediate precision and repeatability. Here, staphylococcal phage K was tested using the double overlay spot assay against 154 periprosthetic joint infection-associated *Staphylococcus aureus* isolates in technical and biological triplicates. In addition to measuring phage titers, plaque-forming units were assessed with a scoring system. Results indicate high intermediate precision and repeatability of the method described.

## INTRODUCTION

Lytic bacteriophages are being evaluated as an alternative or adjuvant to conventional antibiotics for treatment of recalcitrant bacterial infections (1, 2). Despite case reports suggesting efficacy and ongoing clinical trials, phage therapy has not been established as a standard of care in clinical practice (3). Its implementation will require definition of optimal routes of administration, dosing regimens, the role of host immune response against phage, and establishment of standardized phage susceptibility testing (PST) methods (3). With regard to the last, differences in phage-host interactions across bacterial taxa and dependencies of test outcomes on the experimental setup (e.g., media preparation and supplementation) pose hurdles. PST assays should accurately discern nonsusceptible from susceptible isolates, support high-throughput processing, be operationally sustainable for diagnostic laboratories, and have high intermediate precision (i.e., yield the same results when performed on different days—biological replicates) and repeatability (yield the same results when performed contemporaneously—technical replicates), collectively referred to as precision (4). To date, no single method has been developed, validated, and denoted as a reference standard for routine testing (3).

In contrast to antibiotics, for which epidemiological cut-off values provide a rigorous framework to interpret susceptibility, PST lacks such criteria (3). Instead, phage susceptibility can be defined as the ability of a bacterial strain to be infected and lysed, though the phenotypic correlation may be ambiguous as confounding factors, such as “lysis from without,” can occur (3, 5, 6). Several methods leveraging liquid and solid media have been described to characterize phage susceptibility (7–9). With methods using solid media, successful completion of a lytic viral infection cycle typically yields plaque-forming units (PFUs), that is, localized, clear, or translucent zones on bacteria-containing media (7, 9, 10). The double overlay spot assay (i.e., spot assay) is a widely used method to assess PFU on a given host bacterium (3, 11). With a spot assay, plaques may be enumerated (semi-quantitative) and/or categorized (qualitative) to demonstrate phage infectivity (12). Experimental outcomes depend not only on the bacterial host and phage tested but also discrete factors such as top agar composition and preparation, growth phase of host bacteria when plated onto the media, and inclusion of supplements (e.g., MgSO_4_) that can affect phage adsorption (5).

Here, spot assay precision was examined utilizing phage K, a lytic myophage targeting staphylococcal species, against a collection of *Staphylococcus aureus* isolates recovered from patients with periprosthetic joint infection (PJI) (13). A scoring system for qualitative assessment of susceptibility was implemented, and score values correlated with PFU per milliliter (PFU/mL). Collectively, the data generated show that the described PST method has high precision for testing phage K against *S. aureus*.

## MATERIALS AND METHODS

### Bacterial isolates

A total of 154 *S. aureus* PJI isolates collected at the Mayo Clinic, Rochester, MN, between May 1999 and March 2022 were studied. A list of the bacterial isolates, along with all data points utilized for analysis, is provided in Table S1. For each experimental run, *S. aureus* ATCC 19685 (host strain for phage K) served as the positive control, and *Escherichia coli* ATCC 68368 served as the negative control. All isolates were stored at −80°C and retrieved for study by streaking for isolation onto tryptic soy agar (TSA) containing 5% sheep blood (SBA; Becton, Dickinson, Sparks, MD).

### Phage preparation

A high-titer preparation of purified phage K (2 × 10^12±1^ PFU/mL; GenBank accession no. AY176327.1; ATCC 19685-B1), suspended in phage buffer (100 mM NaCl, 10 mM MgCl₂, and 50 mM Tris-HCl; pH 8.0), was obtained from TAILΦR Labs at Baylor College of Medicine (Houston, TX). Purified phage K stocks were protected from light and stored at 4°C until used for downstream propagation, which was done as follows: host bacteria (ATCC 19685) were allowed to reach log phase in tryptic soy broth (TSB, Merck, Darmstadt, Germany). Once a turbidity of 0.5 McFarland was reached, purified phage K stock, resuspended to 10^8^ PFU/mL in phosphate-buffered saline (PBS; Cytiva, Logan, UT), was added at a 1:10 final dilution, corresponding to a multiplicity of infection (MOI) of ~0.1, and the mixture incubated overnight on an orbital shaker at 37°C. Then, the lysate was centrifuged at 5,000 rpm for 5 min at room temperature, filter-sterilized (0.22 µm filter), titered (as described in the Overlay soft agar and Spot assay sections below), and stored at 4°C until further use.

### Overlay soft agar

Soft agar was prepared in 600 mL batches by mixing 18 g of TSB, 4.5 g of agar (Fisher Scientific, Waltham, MA), and 6 mL of 1M MgSO_4_ (Sigma-Aldrich, St. Louis, MO) with deionized water. The mixture was boiled while stirring on a hotplate. Aliquots (5 mL) were dispensed into glass vials (Fisher Scientific, Waltham, MA), and vials were loosely capped and covered with aluminum foil. After autoclaving at 121°C for 20 min, caps were tightened; soft agar was kept at room temperature until further use.

### Spot assay

Experiments were conducted in biological triplicates, with each biological replicate being performed on a different day in technical triplicates (i.e., 9 data points per bacterial isolate). Clinical isolates were cultured on 5% sheep blood agar and grown overnight at 37°C in 5% CO_2_. A single isolated colony was inoculated into 2 mL of TSB and incubated at 37°C shaking at 200 rpm for 16 h. On the day of testing, soft agar vials were melted and molten soft agar vials placed in a heating block at 50°C. Each overnight culture was vortexed at maximum speed for 3 s. Then, 100 µL of the resuspended bacterial culture was transferred into molten soft agar (equilibrated to 50°C), gently mixed by rolling between the palms for 3 s, and poured on a tryptic soy agar plate (Fisher Scientific, Waltham, MA). After the poured bacteria-agar suspension solidified, the phage K lysate was serially diluted (1:10) in PBS and 2 µl aliquots of each dilution spotted onto the solidified soft agar overlay using a multichannel pipette (Fisher Scientific, Waltham, MA). Plates were incubated overnight at 37°C in room air.

### Morphology assessment

**Fig 1:**
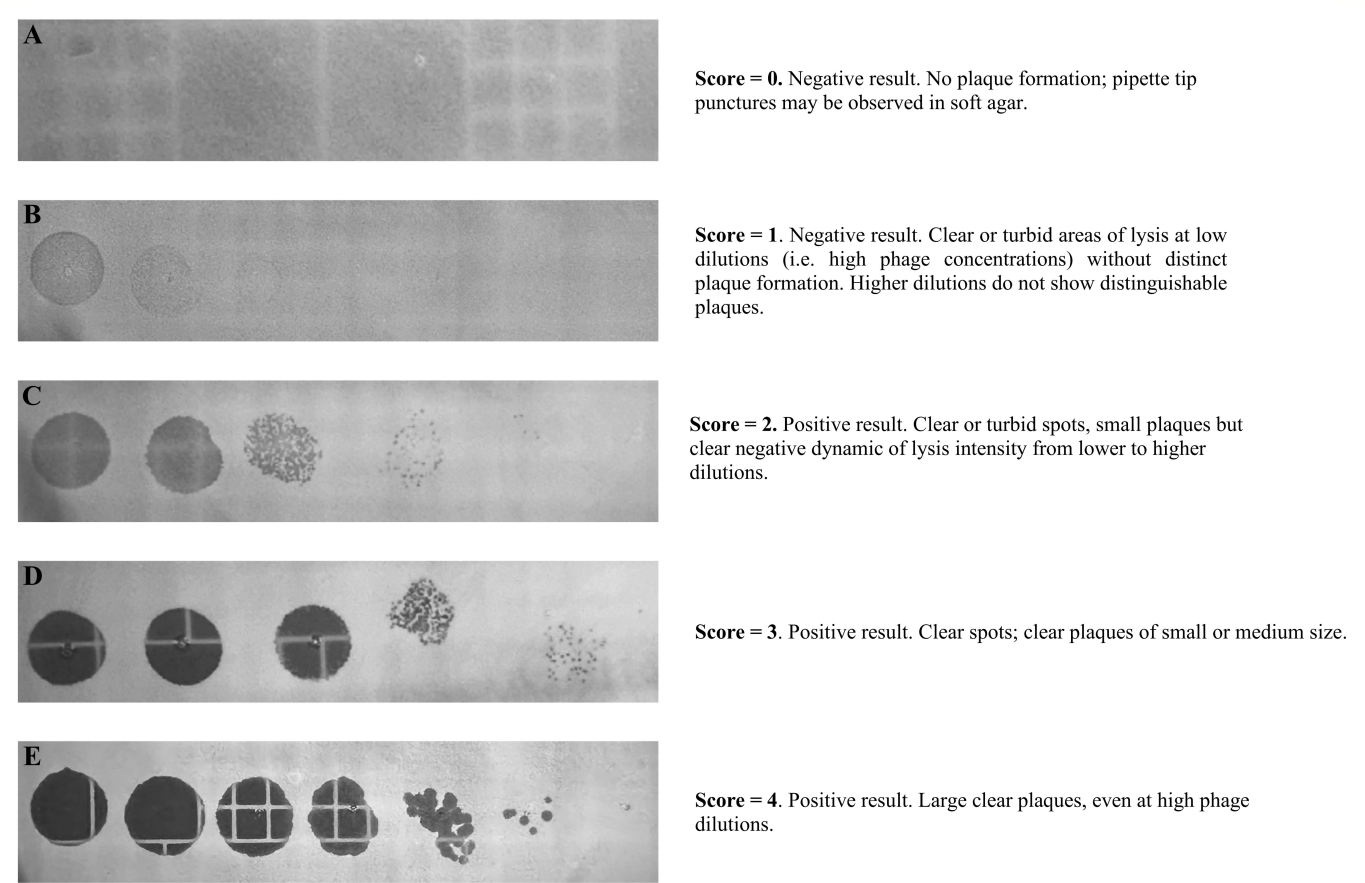
Morphology scores were recorded as 0–4. Successful phage infection (positive result) was defined as a score of ≥2, with lower scores indicating no plaque formation. (A) corresponds to a score of 0 (no infectivity detected, thus denoting a negative result), (B) a score of 1, (C) a score of 2, (D) a score of 3, and (E) a score of 4. A brief description of each score is shown on the right and a representative photograph on the left. Ten-fold dilutions (from 1:10 to 1:10^−8^) were plated from left to right (eighth dilution not shown). The limit of detection for PFU counts was 5,000 PFU/mL.

Spot assay phenotypes were assessed using a scoring system adapted from the Walter-Reed Army Institute of Research, with modification (12). A single operator assigned scores of 0 to 4 based on the clarity, size, and definition of plaques (Fig. 1).

### Quantitative analysis

Quantification was performed for isolates with qualitative scores ≥2. In the absence of plaque formation (i.e., qualitative score 0–1), results were recorded as 0 PFU/mL. However, for data visualization and statistical analysis, isolates with a qualitative score of 0–1 (corresponding to 0 PFU/mL) were assigned a value of 3.7 log_10_ PFU/mL, the limit of detection (LOD) of the assay (corresponding to a single PFU at the lowest dilution tested). PFUs were counted from the highest dilution where at least 10 PFUs were visible. PFU/mL was then calculated using the following:


$$PFU/mL\;=\;\frac{Number\;of\;plaques\;\times \;dilution\;factor}{0.002\;mL}$$

### Statistical analysis

**Fig 2:**
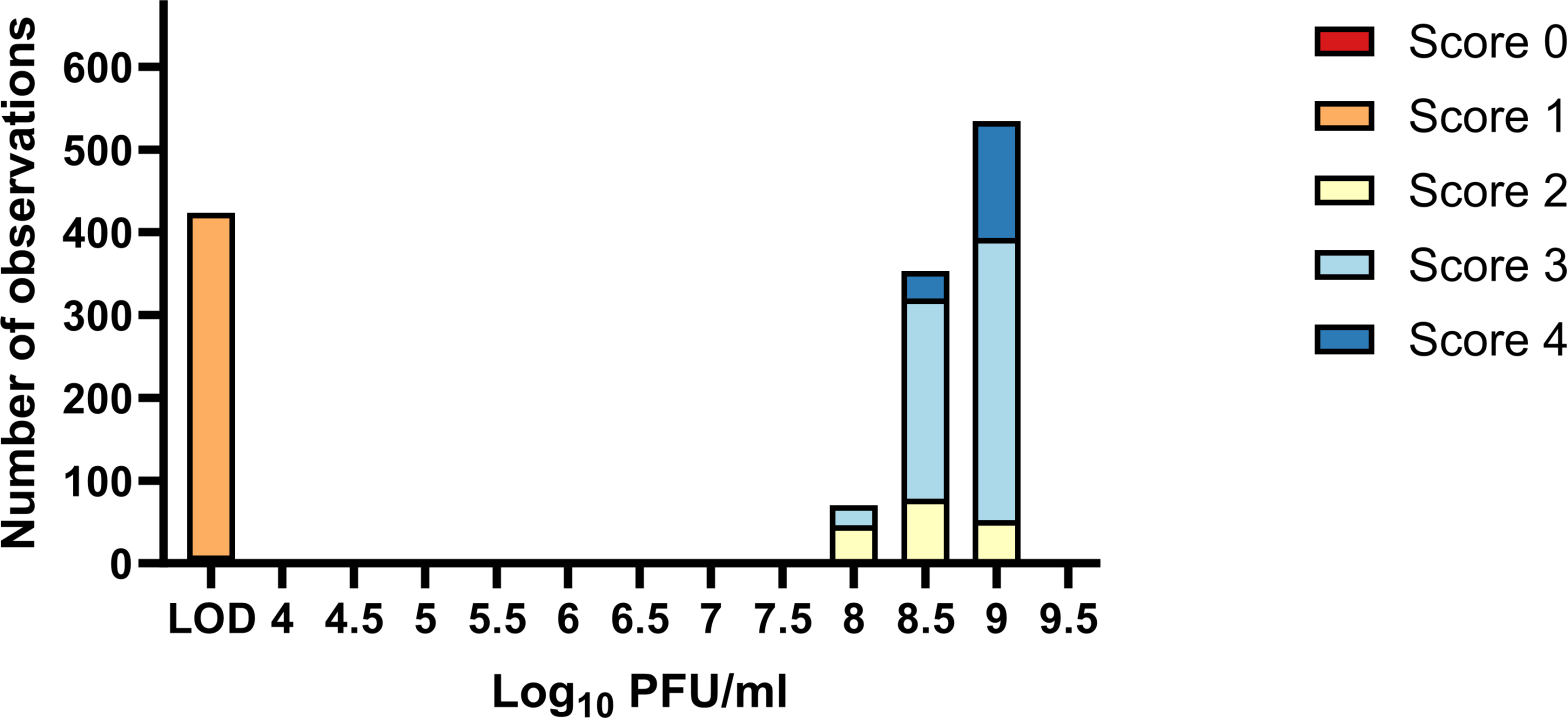
Phage titers (in log_10_ PFU/mL) and respective morphology scores (0–4). Numbers of observations for each log_10_ PFU/mL technical replicate for each isolate are plotted, along with the corresponding morphology score. A score of 0 was observed 9 times at the LOD, which was 3.7 log_10_ PFU/mL. Plaque scores ≥2 were interpreted as successful phage infection.

Statistical analysis was performed using GraphPad Prism (version 10.0.0) and RStudio (version 2025.5.1.513). Initial RStudio source code (to create Fig. 2; Fig. S1 to S3; Table S1) was crafted by ChatGPT (OpenAI, version 5), with subsequent proofreading and adaptation by the authors. Coefficient of variation (CV%) was calculated as the standard deviation divided by the mean, multiplied by 100, for both technical (i.e., contemporaneous) and biological (i.e., inter-day) replicates; CV% values equal to 0 (corresponding to isolates with identical log_10_-transformed titers) were not included when calculating mean CV% to avoid bias. Spearman’s rank correlation (two-tailed) was implemented to assess the correlation between log_10_-transformed titers and morphology scores. For inter-day comparisons, three log_10_ technical replicates were averaged and utilized for pairwise comparisons (day 1 *versus* 2, day 2 *versus* 3, and day 1 *versus* 3) using Pearson’s correlation coefficient (r). All tests were two-sided, and statistical significance was defined as *P* < 0.05 for all tests.

## RESULTS

Testing 154 clinical *S. aureus* PJI isolates yielded a total of 1,386 data points for analysis (Fig. 2). Of the 154 isolates tested, 44 yielded a score of <2 (i.e., negative result) with a log_10_ value at the limit of detection (i.e., 3.7 log_10_ PFU/mL) for all tests. One hundred seven isolates yielded scores ≥2 and log_10_ values above the limit of detection for all tests. Three isolates, IDRL-8893, IDRL-6114, and IDRL-6604, yielded discrepant results (Fig. S3A and B. The 154 isolates were analyzed for consistency across technical and biological replicate plating. The mean CV% was 0.9 among eligible technical replicates (i.e., replicates with non-zero CV%), indicating excellent repeatability. When comparing biological triplicates (i.e., intermediate precision), the CV% was 2.5, with r values of 0.97 (day 1 *versus* 2), 0.98 (day 2 *versus* 3), and 0.96 (day 1 *versus* 3; Fig. S2A through F), indicating high intermediate precision among tested isolates. Spearman’s rank correlation showed a strong positive association between log_10_-transformed titers and morphology scores (ρ = 0.801, *P* < 0.0001, Fig. S1).

## DISCUSSION

Precision is a prerequisite for establishing a clinically useful PST method (3). While many PST methods have been described, no single assay or workflow is considered the reference standard (3). The spot assay has been used by many investigators (14–17). Its suitability across bacterial species varies, with some sources reporting poor reproducibility (3, 16, 17). Therefore, investigations evaluating the appropriateness of this method for any given bacterium and phage are ideal (3). To that end, this study sought to assess spot assay precision utilizing phage K, a well-described staphylococcal phage (18, 19), and clinical *S. aureus* PJI isolates. Assay precision was subgrouped into repeatability (comparing technical replicates) and intermediate precision (comparing biological replicates tested on days).

A total of 154 clinical *S. aureus* isolates were tested in both technical and biological triplicates. In addition to PFU/mL measurements, individual plaques were scored for each isolate (Fig. 1). The CV% for intra- and inter-day analysis was 0.9% and 2.5%, respectively. Furthermore, r^2^ values showed excellent inter-day agreement (≥0.96) for log_10_ PFU/mL values. Collectively, these results suggest the spot assay, as implemented here, produces precise results for clinical *S. aureus* and phage K. This is visualized in Fig. 2, where the bimodal distribution reflects either scores of 0 or 1 with PFU/mL titers at the LOD or scores ≥2 with high PFU/mL titers for any given isolate. Only three isolates (1.95%) yielded discrepant results. Discrepancies found with these three isolates may be due to changes in bacterial physiology rather than spot assay methodology. As a final verification of precision, the positive and negative control strains used throughout the testing of 154 clinical isolates remained concordant in PFU/mL titers and scores (Fig. S4).

The positive correlation of semi-quantitative titers with scores (Spearman’s ρ = 0.8, *P* < 0.0001) may help facilitate testing procedures and be used as a secondary confirmation of infectivity. However, plaque morphology is dependent on the media and supplements used to grow the bacterial strain (5). Here, soft agar was supplemented with MgSO_4_ to enhance phage adsorption (5, 20). MgSO_4_ was added before autoclaving soft agar to streamline media preparation; however, exposure to high temperatures and pressure may have precipitated magnesium salts as minor residual was observed at the bottom of the tubes. Gentle swirling of the molten agar before pouring was used to promote an even distribution. Scale-up of this method may influence plaque quality and thus morphological scoring results. Therefore, consistent media preparation is essential to achieving precise spot assay results.

While the described assay shows promise under the conditions tested, several limitations must be considered. First, the analytical specificity of spot assays to discriminate phage-susceptible from phage-resistant bacteria is inherently limited as phage susceptibility is still possible in the absence of plaque formation, and *vice versa* (5). To that end, the scoring system presented here only assesses plaque formation; scores of 1, consistent with “lysis from without,” are of unclear therapeutic implication. Choosing 2 µL spot volumes simplified plate handling—individual spots were less likely to merge, and expeditious diffusion into the soft agar was possible, but this also meant the LOD was 5,000 PFU/mL. Although not suitable for exact viral quantification, results presented indicate assay precision to a degree where they should permit longitudinal comparisons, for example, when testing serial isolates from the same patient over time. A final limitation is that only one phage and one source of bacterial isolates (PJI) was investigated at a single center by a single investigator.

In conclusion, a spot assay performed on an MgSO_4_-supplemented soft agar overlay yielded results with high precision for phage K tested against 154 *S. aureus* PJI isolates.

## Data Availability

Data are available upon request.

## SUPPLEMENTAL MATERIAL

Supplemental figures spectrum.03284-25-s0001.docx Fig. S1 to S4.

Table S1 spectrum.03284-25-s0002.xlsx Measurement data.

ASM does not own the copyrights to Supplemental Material that may be linked to, or accessed through, an article. The authors have granted ASM a non-exclusive, world-wide license to publish the Supplemental Material files. Please contact the corresponding author directly for reuse.
