# Supplementary material for: Precision of a phage susceptibility spot assay assessed with 154 clinical Staphylococcus aureus isolates
Source: Microbiol Spectr. 2026 Mar 3;14(4):e03284-25. doi: 10.1128/spectrum.03284-25 (PMC13055285; doi:10.1128/spectrum.03284-25)
Supplement: Supplemental figures — Fig. S1 to S4. [file spectrum.03284-25-s0001.docx]

**Supplementary Material**

**
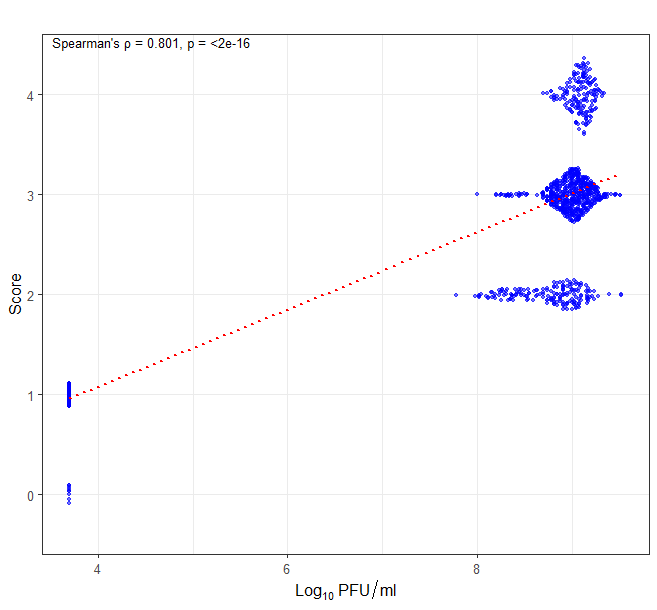
**

**Supplementary Figure 1.** Log_10_ PFU/ml titers and morphology scores, assessed by Spearman’s rank correlation (two tailed), including best fit curve in red. Rank coefficient (ρ) and p-value are indicated in the graph.

**
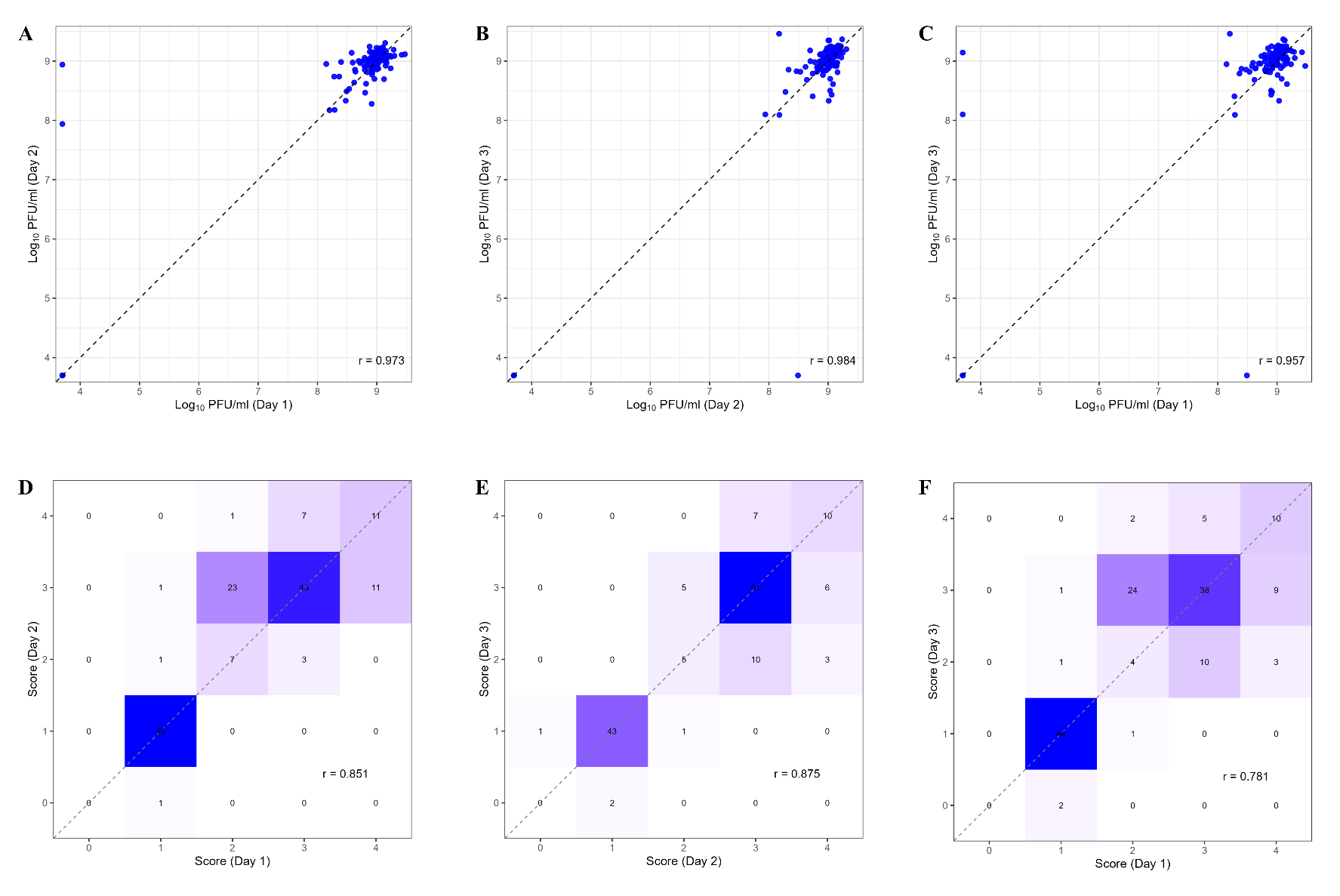
Supplementary Figure 2. Correlation and agreement log_10_ PFU/ml values and morphology scores across experimental days.** Pearson’s correlation coefficient (r) is shown in the bottom corner of each panel. **A, B and C** scatterplots of log_10_ PFU/ml values comparing day 1 *versus* 2, day 2 *versus* 3, and day 1 *versus* 3, respectively. Each dot represents the mean value of a technical triplicate. The dashed lines represent lines of identity. **D, E and F** heatmaps showing morphology scores (0-4) comparing day 1 *versus* 2, day 2 *versus* 3, and day 1 *versus* 3, respectively. The dashed lines indicate perfect agreement.

**
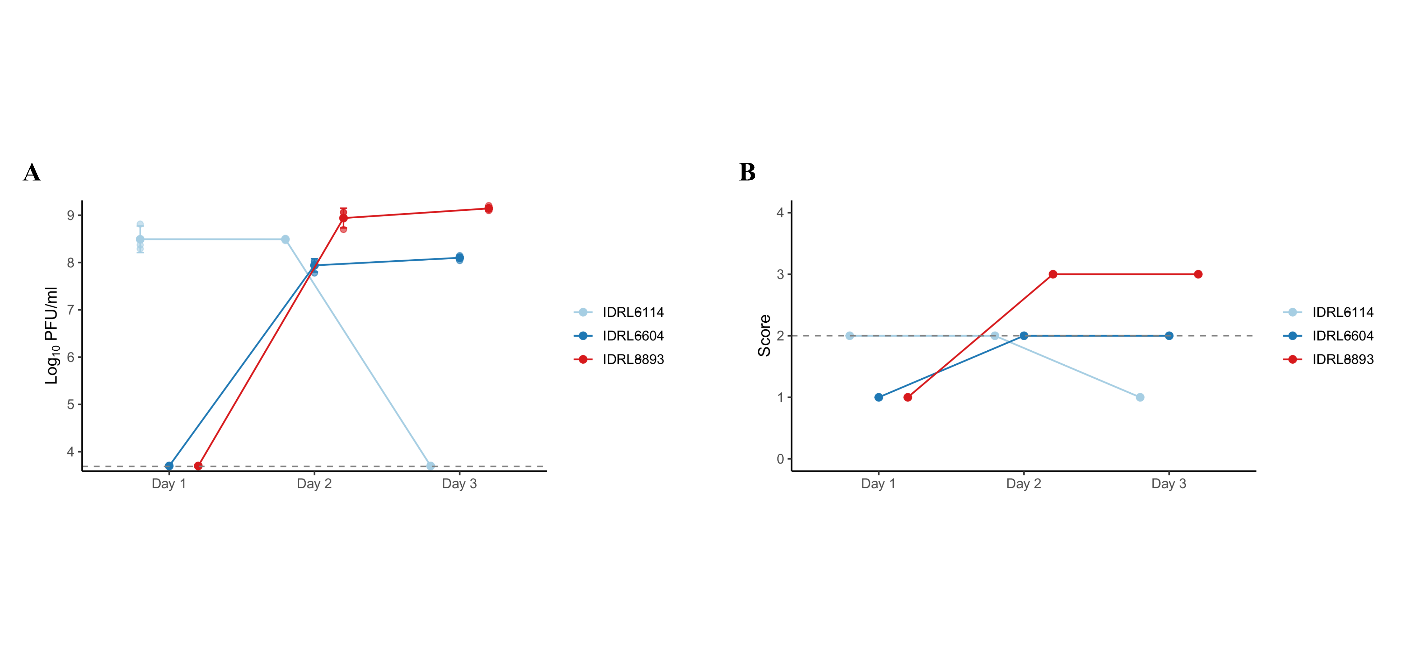
**

**Supplementary Figure 3. log_10_ PFU/ml values and morphology scores for the four discrepant bacterial isolates across experimental days.** Each color represents a different isolate. **A** Log_10_ PFU/ml values of three isolates with inconsistent day-to-day behavior. Individual replicates are shown as semi-transparent points; solid circles show mean values. The dashed line represents the limit of detection. **B** Morphology scores (0-4) of discrepant isolates. The dashed line represents the score cut-off for successful infection (i.e., ≥2).

**
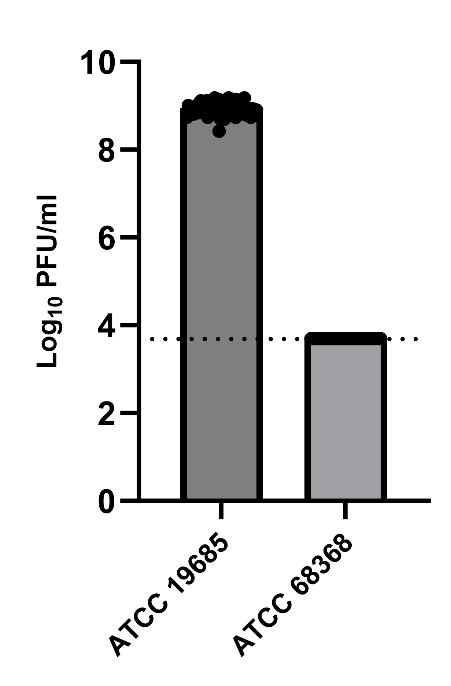
**

**Supplementary Figure 4. Log_10_ PFU/ml values of control strains.** Each experimental run was conducted with a positive control (ATCC 19685, host strain for phage K) and a negative control (ATCC 68368, *Escherichia coli*). PFU/ml measurements and scores (not shown) showed minimal variation across experiments. The dashed line represents the limit of detection (3.7 log_10_ PFU/ml).
